# La dysfonction érectile chez l'hypertendu togolais: étude transversale chez 100 patients dans le Service de Cardiologie du CHU Campus de Lomé

**DOI:** 10.11604/pamj.2015.21.47.5401

**Published:** 2015-05-22

**Authors:** Soulemane Pessinaba, Soodougoua Baragou, Machihude Pio, Kevin Tengué, Yaovi Afassinou, Matchona Kpatcha, Madikizi Awisoba, Mouhamed Kpélafia, NKenon Watani Nda, Findibé Damorou

**Affiliations:** 1 Service de Cardiologie, CHU Campus, 03BP: 30284, Lomé, Togo; 2 Service de Cardiologie, CHU Sylvanus Olympio, Lomé, Togo; 3 Service d'Urologie, CHU Sylvanus Olympio, Lomé, Togo

**Keywords:** Dysfonction érectile, HTA, prévalence, sévérité, erectile dysfunction, HBP, prevalence, severity

## Abstract

**Introduction:**

L'association HTA et dysfonction érectile (DE) est connue. Les objectifs de ce travail étaient de déterminer la prévalence de la DE et d'en évaluer la sévérité chez l'hypertendu togolais.

**Méthodes:**

Il s'agit d'une étude transversale menée chez 100 hypertendus reçus en consultations externes dans le service cardiologie du CHU Campus d'octobre à décembre 2012. Le statut érectile a été évalué avec l'international index of erectile function (IIEF-5).

**Résultats:**

L’âge moyen des hypertendus était de 53,3 ± 10,3 ans. La durée moyenne de l'HTA était de 6,7 ± 6,9 ans. Les facteurs de risque cardio-vasculaire retrouvés chez ces hypertendus étaient: diabète (7%), obésité (17,5%), obésité abdominale (40%), dyslipidémie (36%) et tabac (1%). La prévalence de la DE était de 53% dont 32% de DE légère, 18% de DE modérée et 3% de DE sévère. Parmi les patients ayant une DE, 60% avaient une DE légère, 34% une DE modérée et 6% une DE sévère. Le score IIFE moyen était de 19,1 ± 5,2. La prévalence de la DE augmentait avec l’âge (p=0,05), le grade (p=0,004) et la durée de l'HTA (p=0,20). La DE était significativement plus fréquente en présence du diabète (p=0,02) et de l'obésité abdominale (p=0,007).

**Conclusion:**

La prévalence de la DE est élevée chez l'hypertendu togolais. Cette prévalence augmente avec l’âge, la durée de l'HTA, la présence de diabète et l'obésité abdominale. La DE doit être systématiquement recherchée chez l'hypertendu surtout en présence des autres FDR.

## Introduction

La dysfonction érectile (DE) est une pathologie fréquente. Sa prévalence était de 52% chez les hommes âgés de 40 à 70 ans dans l’étude “Massachusetts Male Aging Study” [1]. En 1995, on estimait que la DE affectait 152 millions d'hommes dans le monde. En 2025, elle toucherait 322 millions d'hommes avec une large augmentation dans les pays en développement notamment en Afrique, Asie et Amérique du Sud [2]. L'hypertension artérielle (HTA), un facteur de risque indépendant de DE touche actuellement environ 27 à 28% de la population adulte âgée de 20 ans et plus en Afrique subsaharienne; environ 80 millions de patients souffraient d'HTA en 2000, et selon les projections épidémiologiques ils seront 150 millions en 2025 [3, 4]. Au Togo, l'HTA représentait 36,7% dans la population générale à Lomé [5] et 74,29% des admissions dans le service de cardiologie du CHU Campus chez les sujets de plus de 50 ans [6]. L'association HTA et DE est bien connue. A l'inverse, les traitements anti-hypertenseurs peuvent favoriser la survenue d'une dysfonction érectile, ce qui rend parfois difficile l'observance thérapeutique des hypertendus. La prévalence de la DE est significativement plus élevée chez les hommes hypertendus que dans la population générale [7–9]. Les objectifs de ce travail étaient de déterminer la prévalence de la DE et d'en évaluer la sévérité chez l'hypertendu togolais.

## Méthodes

Nous avons mené une étude transversale et descriptive chez 100 patients reçus en consultations externes dans le service cardiologie du CHU Campus d'octobre à décembre 2012 pour HTA. Ont été inclus les patients connus hypertendus depuis au moins un an et ayant donné un consentement verbal à participer à l’étude. Les critères d'exclusion avaient été définis comme suit: antécédents de dysfonction myocardique documentée avec une fraction d’éjection du ventricule gauche inférieure à 50%; antécédents de chirurgie pelvienne, urologique ou colorectale; antécédents d'irradiation pelvienne et/ou antécédents de pathologie neurologique de quelque nature que ce soit; et le refus de participer à l’étude

Pour chaque patient, les données suivantes ont été relevées: l'ancienneté, la sévérité de l'HTA et traitement en cours; l’âge, le poids, l'indice de masse corporelle (IMC) et le périmètre abdominal (PA); l'existence d'autres facteurs de risque cardiovasculaires tels que le tabagisme, la dyslipidémie, le diabète et l'obésité.

Pour évaluer le statut érectile, nous avons utilisé le questionnaire *international index of erectile function* (IIEF-5) [10]. Après dépouillement des questionnaires, l'intensité de la DE était finalement répertoriée de façon objective selon quatre classes: « pas de DE » entre 21 et 25 inclus, «DE légère» inférieure à 21, « DE modérée » inférieure à 17 et «DE sévère» inférieure à 10 [11]. L'analyse statistique des données a été faite à l'aide du logiciel Epi info dans sa version 3.5.1. Le test de khi2 ou Fisher exact a été utilisé pour comparer les proportions et le test de Student pour la comparaison des moyennes. Une valeur de *p* < 0,05 à a été considéré comme statistiquement significative.

## Résultats

**Figure 1:**
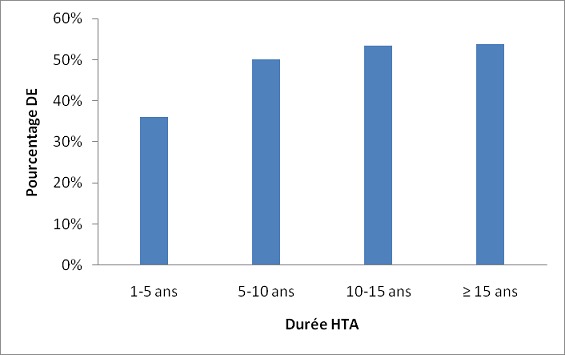
Répartition des patients en fonction de la durée d’évolution de l'hypertension artérielle

**Figure 2:**
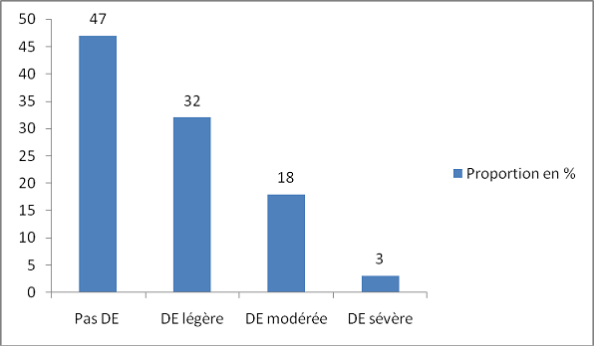
Répartition des patients en fonction du score de l'IIFE

**Figure 3:**
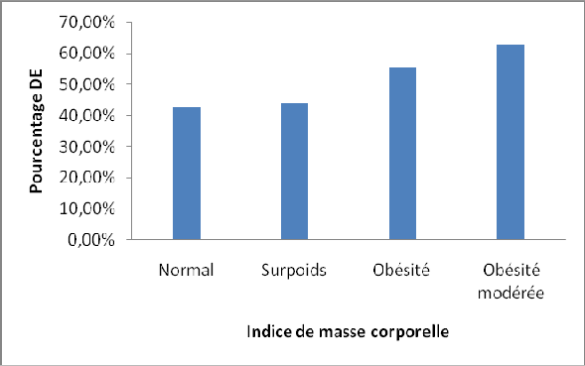
Répartition de la dysfonction érectile en fonction de la l'indice de masse corporelle

L’âge moyen des 100 patients était de 53,3 ± 10,3 ans avec des extrêmes de 30 et 78 ans. Les tranches de 40 à 50 ans et de 50 à 60 ans étaient les plus représentées. Le Tableau 1 regroupe les caractéristiques générales des patients. Sur les 100 patients, 98 étaient mariés et 02 étaient célibataires. La durée moyenne d’évolution de l'HTA était de 6,7± 6,9 ans (extrêmes: 1 et 33 ans). La moitié des patients avait une durée d'HTA entre 1 et 5 ans. La Figure 1 illustre la répartition de la population en fonction de la durée de la maladie. En fonction de la sévérité de l'HTA les proportions étaient suivantes: 6% Pression artérielle normalisée, 37% (HTA grade I), 35% (HTA grade II) et 22% (HTA grade III). Les autres facteurs de risque cardiovasculaire sont représentés dans le Tableau 2. Après étude et analyse du questionnaire rempli par les patients, nous avons obtenus les résultats représentés sur la Figure 2. Au total, 53% des patients présentaient une DE. La DE légère et la DE modérée représentent la moitié de la population. Parmi les patients ayant une DE: 60% avaient une DE légère, 34% une DE modérée et 6% une DE sévère. Le score IIFE moyen était de 19,3 ± 4,8 avec un minimum de 1 et 25. Ce score IIFE diminuait de façon progressive avec l’âge (p = 0,05), le grade de l'HTA (p = 0,004) et avec la durée de l'HTA (p = 0,20). La prévalence de la DE augmentait avec la durée d’évolution et le grade de l'HTA et au fur et à mesure que l’âge des patients augmentait (Tableau 3). L'HTA sévère était retrouvé dans 14% des cas en l'absence de DE, dans 25% des cas de DE légère, dans 27,8% des cas de DE modérée et dans 100% des cas de DE sévère. En somme tous les cas de DE sévère avaient une HTA grade III. La présence du diabète ou de l'obésité en plus de l'HTA augmentait significativement la prévalence de la DE. Le Tableau 4 montre les proportions de la DE en présence ou non des autres facteurs de risque. Chez les patients diabétiques, 14,3% ne présentaient pas de DE, 42,9% présentaient une DE légère, 14,3% présentaient une DE modérée et 28,6% présentaient une DE sévère. La prévalence de la DE augmentait avec la valeur de l'IMC comme le montre la Figure 3.

**Tableau 1:** Caractéristiques générales des patients

Paramètres	moyenne	minimal	maximal
Âge (ans)	53,3 ± 10,3	30	78
Poids (kg)	76,9 ± 13,5	53	123
Taille (m)	1,72 ± 0,71	1,56	1,92
Indice de masse corporelle (kg/m^2^)	25,9 ± 3,8	18,3	35,8
Périmètre abdominal (cm)	90,9 ± 12,8	58	138

**Tableau 2:** Prévalence des autres facteurs de risque cardiovasculaire

Facteurs de risques	Proportion en%
Diabète	7
Tabagisme	1
Dyslipidémie	36
Obésité	17,5
Obésité abdominale (PA > 102 cm)	15
Obésité abdominale (PA >94 cm)	40

PA : périmètre abdominal

**Tableau 3:** Prévalence de la dysfonction érectile et l'IIFE moyen en fonction de la tranche d’âge, du grade et de la durée d’évolution de l'HTA

Variables	DE (%)	p	IIFE moyen	p
**Tranches d’âge**				
30 - 40 ans	23,1		20,7 ± 3,4	
40 - 50 ans	38,2		19,8 ± 3,5	
50 - 60 ans	60		19,5 ± 4,9	0,15
60 - 70 ans	60,9		17,6 ± 5,5	
≥ 70 ans	75		17,3 ± 6,2	
**Grade HTA**				
Grade I	45,7		20,9 ± 3,1	
Grade II	51,4		19,3 ± 5,5	0,004
Grade III	72,7		16,3 ± 7,3	
**Durée d'HTA**				
1 - 5 ans	36		20,2 ± 4,5	
5 - 10 ans	50		18,7 ± 5,7	
10 - 15 ans	53,3		18,6 ± 3,9	0,41
≥ 15 ans	53,8		18,4 ± 5,6	

DE : dysfonction érectile; HTA : hypertension artérielle; IIFE : index international de la fonction érectile

**Tableau 4:** Prévalence de la DE en fonction de présence ou non des autres facteurs de risque cardio-vasculaire

		Dysfonction érectile		p
		Oui	Oui	
Diabète	Oui	85,7%	14,3%	0,02
	Non	40,9%	59,1%	
Dyslipidémie	Oui	61,1%	38,9%	0,12
	Non	46,9%	53,1%	
Périmètre abdominal > 102 cm	Oui	66,7%	33,3%	0,05
	Non	40%	60%	
Périmètre abdominal > 94 cm	Oui	60%	40%	0,007
	Non	33,4%	66,7%	

## Discussion

Cette étude a été réalisée en vue d’évaluer la prévalence de la DE qui est souvent ignorée par le corps médical malgré son importance et son impact sur la qualité de vie des patients. Au terme de notre travail, nous pouvons évoquer les difficultés suivantes: la pudeur: les questions étaient basées sur la vie sexuelle de nos patients, ce qui quelquefois les embarrassaient et pourraient influencer leur réponse; la nature du questionnaire: un questionnaire avec des propositions fermées ne trouve pas toujours d’équivalence avec les réponses des patients; la non maitrise parfaite du français chez certains de nos patients: ce qui a pu créer des biais d'interprétation.

Néanmoins, cette étude présente également des intérêts car elle est la première du genre réalisée en milieu hospitalier au Togo. Et elle s'est dotée d'un questionnaire internationalement reconnu pour l’évaluation et l'appréciation de la fonction érectile des hommes. La prévalence de la DE dans notre population était de 53%. Cette prévalence se situe dans la fourchette de celles retrouvées dans d'autres études chez les hypertendus. Au Qatar, la prévalence de la DE était de 66,2% chez 296 hypertendus [12]. Guiliano et al. en France retrouvaient 61% chez 3906 patients hypertendus [7]. D'autres études avaient retrouvé des prévalences légèrement inférieures à la notre; notamment en Egypte dans une étude multicentrique nationale la prévalence était de 43,2% [13] quand elle était de 45,8% dans une autre étude multicentrique espagnole incluant 2130 hypertendus [14]. Ces études varient dans leur qualité, car elles utilisent différentes définitions de la DE, différentes réponses aux questionnaires et interviews, des disparités culturelles pour discuter des problèmes sexuels et des interprétations différentes des résultats. Malgré les différences dans les prévalences de la DE érectile chez les hypertendus, la plupart des études ont montré une prévalence plus élevée chez les hypertendus que les normotendus. Dans une autre étude réalisée par Cordier et al. chez les patients insuffisant coronariens, la prévalence de la DE était de 0,82 avant la survenue de l’événement coronariens [15]. Ceci confirme le fait que la DE érectile un important marqueur du risque vasculaire.

Comme cela a été retrouvé par Bener et al. au Qatar [12], la prévalence de la DE augmentait avec la durée et la sévérité de l'HTA dans notre travail. Ceci traduit l'impacte de l'HTA sur l'altération de la fonction érectile. De même, le score moyen de l'IIFE était significativement plus bas dans notre travail comme dans celui de Bener et al. [12]. La prévalence de la DE augmentait avec l’âge de nos patients hypertendus. Ce même résultat a été noté dans les travaux réalisés au Qatar et en Espagne [12, 13]. Ce constat n'est pas particulier aux hypertendus puisse que des études en population générale ou chez les sujets normotendus montrent également une corrélation entre l’âge et la DE [16]. Le diabète, l'obésité, les dyslipidémies et le tabac sont connus comme étant de puissants facteurs de risque cardiovasculaire. La présence du diabète, de la dyslipidémie et de l'obésité chez nos patients hypertendus augmentait significativement la prévalence de la DE. Cet effet délétère et synergique de ces facteurs de risque sur la fonction érectile a été retrouvé dans plusieurs d'autres études [8, 12]. Le taux de tabagisme faible dans notre étude ne permettait d'analyser sa relation avec la DE.

## Conclusion

La prévalence de la DE est élevée chez l'hypertendus togolais. La DE est le plus souvent légère à modérée et est d'autant plus sévère que le garde de l'HTA augmente. Cette prévalence augmente avec l’âge, la durée d’évolution et le grade de l'HTA; et avec la présence des autres facteurs de risque cardiovasculaire. Il s'avère donc important de rechercher systématiquement une DE chez nos patients hypertendus surtout lorsqu'il s'agit d'une HTA sévère et/ou associée aux autres facteurs de risque cardiovasculaire.
